# Synthesis of bis(3-{[2-(allyloxy)ethoxy]methyl}-2,4,6-trimethylbenzoyl)(phenyl)phosphine oxide – a tailor-made photoinitiator for dental adhesives

**DOI:** 10.3762/bjoc.6.26

**Published:** 2010-03-15

**Authors:** Norbert Moszner, Iris Lamparth, Jörg Angermann, Urs Karl Fischer, Frank Zeuner, Thorsten Bock, Robert Liska, Volker Rheinberger

**Affiliations:** 1 Ivoclar Vivadent AG, Bendererstrasse 2, FL-9494 Schaan, Liechtenstein; 2 Institute of Applied Synthetic Chemistry, Division of Macromolecular Chemistry, Vienna University of Technology, Getreidemarkt 9/163/MA, A-1060 Vienna, Austria

**Keywords:** adhesives, dental polymers, dimethacrylates, photoinitiator, radical polymerization

## Abstract

Because of the poor solubility of the commercially available bisacylphosphine oxides in dental acidic aqueous primer formulations, bis(3-{[2-(allyloxy)ethoxy]methyl}-2,4,6-trimethylbenzoyl)(phenyl)phosphine oxide (WBAPO) was synthesized starting from 3-(chloromethyl)-2,4,6-trimethylbenzoic acid by the dichlorophosphine route. The substituent was introduced by etherification with 2-(allyloxy)ethanol. In the second step, 3-{[2-(allyloxy)ethoxy]methyl}-2,4,6-trimethylbenzoic acid was chlorinated. The formed acid chloride showed an unexpected low thermal stability. Its thermal rearrangement at 180 °C resulted in a fast formation of 3-(chloromethyl)-2,4,6-trimethylbenzoic acid 2-(allyloxy)ethyl ester. In the third step, the acid chloride was reacted with phenylphosphine dilithium with the formation of bis(3-{[2-(allyloxy)ethoxy]methyl}-2,4,6-trimethylbenzoyl)(phenyl)phosphine, which was oxidized to WBAPO. The structure of WBAPO was confirmed by ^1^H NMR, ^13^C NMR, ^31^P NMR, and IR spectroscopy, as well as elemental analysis. WBAPO, a yellow liquid, possesses improved solubility in polar solvents and shows UV–vis absorption, and a high photoreactivity comparable with the commercially available bisacylphosphine oxides. A sufficient storage stability was found in dental acidic aqueous primer formulations.

## Introduction

Self-etching enamel-dentin adhesives (SEAs) are used in restorative dentistry to achieve a strong bond between the filling composites and dental hard tissues. The main components of currently used SEAs include strongly acidic adhesive monomers, such as polymerizable phosphonic or phosphoric acids and crosslinking dimethacrylates, such as 2,2-bis[(4-(2-hydroxy-3-methacryloyloxypropoxy)phenyl]propane (Bis-GMA) or triethylene glycol dimethacrylate (TEGDMA) [1]. In SEAs, water is primarily used as the solvent or co-solvent. Thus, especially in the case of one-bottle adhesives, the methacrylates may undergo hydrolysis of the methacrylate ester groups in the presence of the strongly acidic adhesive monomers. Therefore, we have synthesized new crosslinkers [2], such as *N*,*N*'-diethyl-1,3-bis(acrylamido)propane (DEBAMP), or new strongly acidic monomers, such as 2,4,6-trimethylphenyl 2-[4-(dihydroxyphosphoryl)-2-oxa-butyl]acrylate [3] or 1,3-bis(methacrylamido)propane-2-yl dihydrogen phosphate (BMAMHP) [4], which show improved hydrolytic stability under acidic aqueous conditions. The visible-light (VL) photoinitiators (PIs) in current SEAs are based on mixtures of camphorquinone (CQ) and tertiary amines (A) [5]. The CQ-A PIs belong to bimolecular hydrogen abstraction PI systems: CQ shows a broad absorption spectrum between 400 and 500 nm (λ_max_ = 468 nm). The VL-excited CQ forms an excited state complex with the amine co-initiator, which generates a ketyl and an α-aminoalkyl radical by electron and subsequent proton transfer. The aminoalkyl radical may initiate the polymerization of the monomers present, while the ketyl radical is mainly deactivated by dimerization or disproportionation [6]. However, in SEAs, the acid-base reaction of acidic monomers with the basic amine co-initiators of the PI system may significantly impair the formation of initiating radicals. Moreover, especially in the aqueous medium, the polar radical ions are well solvated by the surrounding medium, thus inhibiting the proton transfer. If proton transfer occurs, both non-ionic and therefore rather hydrophobic species are kept in the solvent cage, which reduces the photoinitiating activity [7]. Therefore, in order to improve the performance of SEAs, amine-free PIs were developed. In this context, we were able to show that benzoyltrimethylgermane [8] and dibenzoyldiethylgermane (DBDEG) [9–10] can be used as VL PIs for the photopolymerization of dimethacrylate resins, dental adhesives or composites and undergo an α-cleavage with the formation of benzoyl and germyl radicals, which may initiate the free-radical polymerization of the monomers present. In addition, bisacylphosphine oxides, such as commercially available bis(2,4,6-trimethylbenzoyl)phenylphosphine oxide (BAPO), seem to be a suitable alternative because their absorption tails out into the visible range of the spectrum [7]. BAPO undergoes a monomolecular α-cleavage with the formation of two initiating radicals and shows a high photoinitiating reactivity with a good storage stability. Recently, a simple straightforward synthesis of BAPO was published [11]. However, the solubility of BAPO in polar solvents and aqueous formulations is insufficiently low, which limits the use of BAPO in water-based SEAs. In this context, we were able to synthesize a number of new substituted bisacylphosphine oxides, which show improved solubility in aqueous compositions [12].

**Scheme 1:**
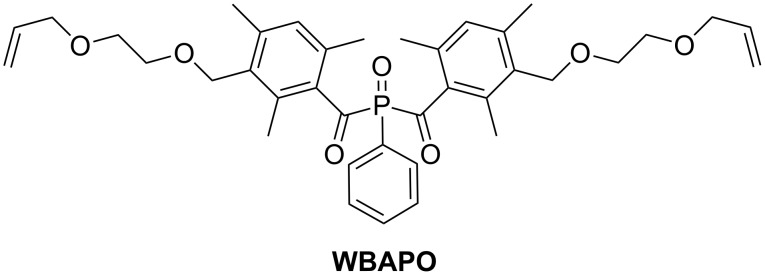
Bisacylphosphine oxide with improved solubility in polar solvents.

In this paper, we report the detailed synthesis of bis(3-{[2-(allyloxy)ethoxy]methyl}-2,4,6-trimethylbenzoyl)(phenyl)phosphine oxide WBAPO (Scheme 1) and its use as a tailor-made PI in SEAs.

## Results and Discussion

### Synthesis and characterization of WBAPO

**Scheme 2:**
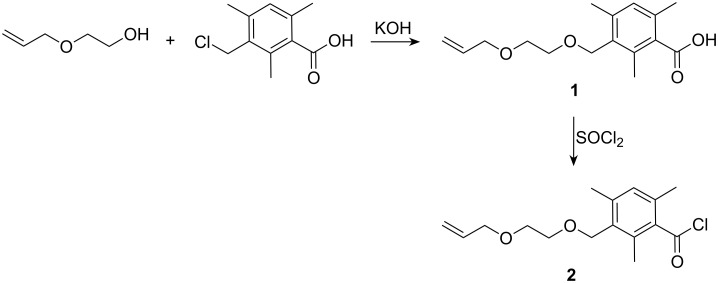
Etherification of 3-(chloromethyl)-2,4,6-trimethylbenzoic acid and chlorination of **1**.

The synthesis of bisacylphosphines and their oxides often start either from *P*,*P*-dichlorophenylphosphine (PhPCl_2_) [13] or free phenylphosphine [14]. We chose to use the PhPCl_2_-route since primary phosphines have an unpleasant smell, a high toxicity and are very air-sensitive. Furthermore, phenylphosphine is difficult to access commercially and is very expensive. The synthesis of WBAPO started from 3-(chloromethyl)-2,4,6-trimethylbenzoic acid, which was synthesized by chloromethylation of 2,4,6-trimethylbenzoic acid with a mixture of paraformaldehyde and hydrochloric acid, followed by treatment of the formed hydroxymethyl compound with concentrated hydrochloric acid in a one-pot reaction [15]. In the first step of the WBAPO synthesis (Scheme 2), 3-(chloromethyl)-2,4,6-trimethylbenzoic acid was coupled with 2-(allyloxy)ethanol using potassium hydroxide in excess of 2-(allyloxy)ethanol as solvent at 70 °C to afford 3-{[2-(allyloxy)ethoxy]methyl}-2,4,6-trimethylbenzoic acid **1** as an off-white powder in 62% yield. The main problem of this step was the separation of the large amount of residual 2-(allyloxy)ethanol. This was successfully accomplished by washing its toluene solution with water followed by recrystallization of the crude product from cyclohexane.

**Scheme 3:**
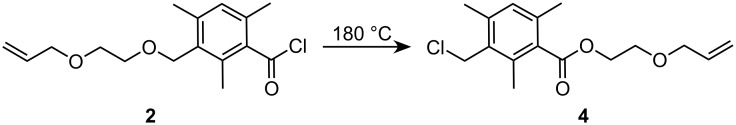
Rearrangement of **2** under formation of **4**.

In the second step, **1** was chlorinated with thionyl chloride in toluene as solvent by a procedure analogous to that described in the literature [16]. After distillation of the dark crude product, 3-{[2-[allyloxy)ethoxy]methyl}-2,4,6-trimethylbenzoyl chloride **2** was obtained as a colorless liquid in 76% yield. However, **2** showed unexpected limited thermal stability. It was found that **2** decomposed with the formation of 3-(chloromethyl)-2,4,6-trimethylbenzoic acid 2-(allyloxy)ethyl ester **4** slowly on storage at room temperature over a few days or rapidly on heating at 180 °C for 4 h. Obviously, this reaction is a thermal rearrangement because an exchange of chlorine with the 2-(allyloxy)ethoxy group took place as a result of heating (Scheme 3). In this context, it should be noted that we also found the exchange of chlorine in the cases of both the 2-(ethoxy)- and 2-(propoxy)ethoxy derivatives. For the successful purification of **2** by distillation both a short distillation time and a relatively low bottom temperature are crucial. Furthermore, it was found to be advantageous if the crude product was purged with nitrogen gas prior to distillation. The identity of compound **4** was established by an independent synthesis of the compound from 2-(allyloxy)ethanol and 3-(chloromethyl)-2,4,6-trimethylbenzoyl chloride **3**, prepared by chlorination of 3-(chloromethyl)-2,4,6-trimethylbenzoic acid with thionyl chloride.

**Scheme 4:**
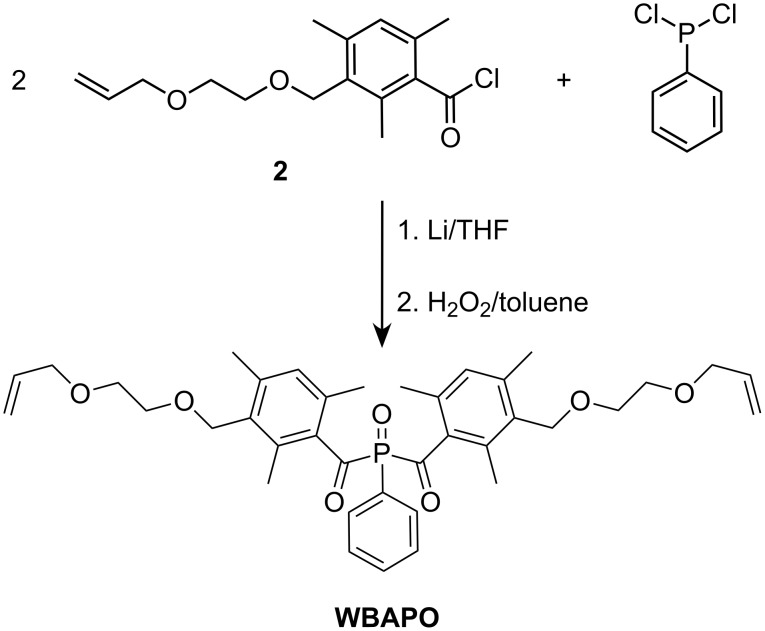
Synthesis of WBAPO starting from *P*,*P*-dichlorophenylphosphine and **2**.

**Figure 1:**
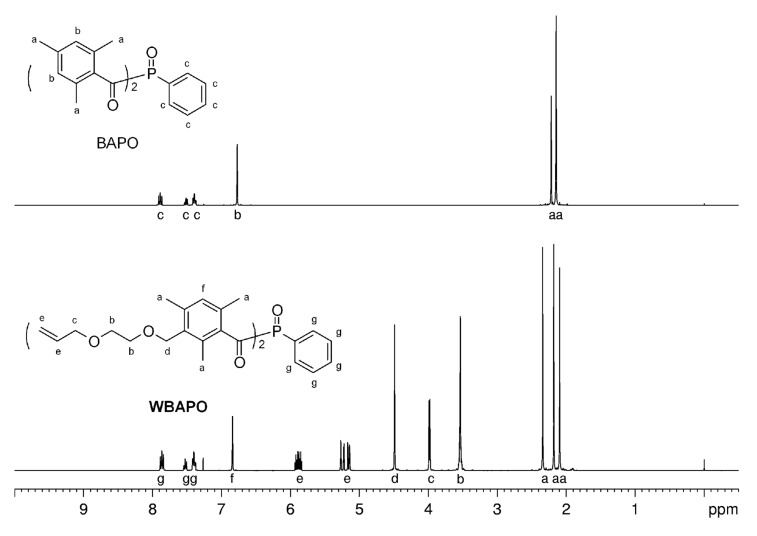
^1^H NMR spectra (400 MHz, CDCl_3_) of BAPO and WBAPO.

In the third step, the bisacylphosphine oxide WBAPO was prepared by the reaction of **2** with *P*,*P*-dichlorophenylphosphine (Scheme 4). Thus, *P*,*P*-dichlorophenylphosphine dissolved in THF was first lithiated with metallic lithium in a dry argon atmosphere in the presence of a small amount of naphthalene. The resulting dark green phenylphosphine dilithium solution was then added to a THF solution of **2**. THF was evaporated from the intermediate bis(3-{[2-(allyloxy)ethoxy]methyl}-2,4,6-trimethylbenzoyl)(phenyl)phosphine solution, the residue dissolved in toluene and oxidized with a 30 wt % hydrogen peroxide solution at 70 °C. After column chromatography, WBAPO was obtained as a yellow oil in 67% yield with a HPLC purity of 94–95%. The characterization of WBAPO was carried out by ^1^H NMR, ^13^C NMR, ^31^P NMR and IR spectroscopy, as well as by elemental analysis. The spectral data are in agreement with the expected structure. For example, the presence of the [2-(allyloxy)ethoxy]methyl substituent was supported by the presence of new signals compared to the spectrum of BAPO: a singlet for the benzyl protons at δ = 4.49 ppm and two multiplets for the vinyl protons at δ = 5.13–5.27 and 5.83–5.93 ppm were evident in the ^1^H NMR spectrum (Figure 1). The ^13^C NMR spectrum of WBAPO showed a doublet arising from the carbonyl carbon atom at δ = 216.2 ppm compared to 216.1 ppm in the case of BAPO, and the ^31^P NMR spectrum featured only one signal at δ = 6.58 ppm (BAPO: 6.98 ppm).

The yield of the WBAPO synthesis was significantly lower compared to the high yield (>90%) in the case of the less substituted bisacylphosphine oxide BAPO. Therefore, a number of model reactions were carried out to elucidate possible reasons for this. We therefore investigated the formation of BAPO by the reaction of 2,4,6-trimethylbenzoylchloride with *P*,*P*-dichlorophenylphosphine under analogous conditions. The lithiation step was carried out in the presence of different model compounds for the reactive sites of the [2-(allyloxy)ethoxy]methyl substituent, such as benzyl methyl ether, allyl ethyl ether or diethylene glycol dimethyl ether. In the case of benzyl methyl ether and allyl ethyl ether, BAPO was formed in a lower yield showing that benzyl and allyl groups had a negative effect on the yield of BAPO. Probably, these groups initiate side reactions during the lithiation step. Furthermore, it was found that the temperature of the phosphine oxidation significantly influenced the yield and the purity of the formed phosphine oxide as it proved very difficult to separate the residual phosphine from the corresponding phosphine oxide. Accordingly, we used temperatures of 45, 50 or 56 °C and determined the area ratios of the WBAPO peak to the peak of the corresponding phosphine. These were found to be 3:5, 4:1 or 8:1 by HPLC. Only temperatures higher than 60 °C ensure a fast and complete oxidation of the bisacylphosphine.

**Scheme 5:**
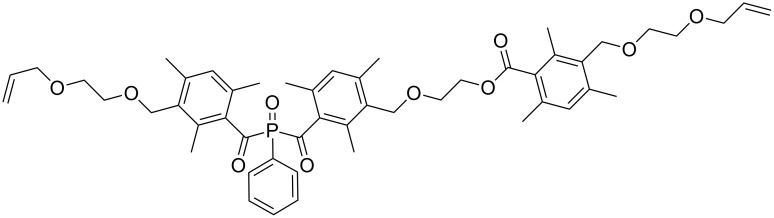
Structure of the main impurity in isolated WBAPO.

Finally, different batches of WBAPO were investigated by LC-MS during the scale-up of the synthesis. The results show that a side-chain extended WBAPO with a molecular weight of 867 g/mol (Scheme 5) was formed in all cases as the main impurity in amounts of 0.5–5%. The structure of this compound was also clearly confirmed by the ^13^C and ^1^H NMR spectroscopic measurements of a WBAPO sample that specifically contained about 70% of this side product. The ^13^C NMR spectrum of this sample showed three carbonyl signals: a singlet at δ = 170.4 ppm and two doublets at δ = 215.9 and 216.5 ppm. The singlet arises from the carbonyl group of the ester, whereas the two doublets can be assigned to the carbonyl groups of the differently substituted and therefore non-equivalent benzoyl moieties. Moreover, in the ^1^H NMR spectrum the signal intensities of CH_3_, =CH (aromat), CH_2_ (benzyl) and CH_2_O protons were increased compared to pure WBAPO. In addition, a new triplet assignable to the CO-O-CH_2_ protons was found at δ = 4.40 ppm. The complete separation of this side compound by repeated column chromatography would be very difficult and expensive. However, it will probably show similar photochemical properties compared to WBAPO and therefore its separation is not necessary.

### Properties of WBAPO, photopolymerization and adhesives

**Figure 2:**
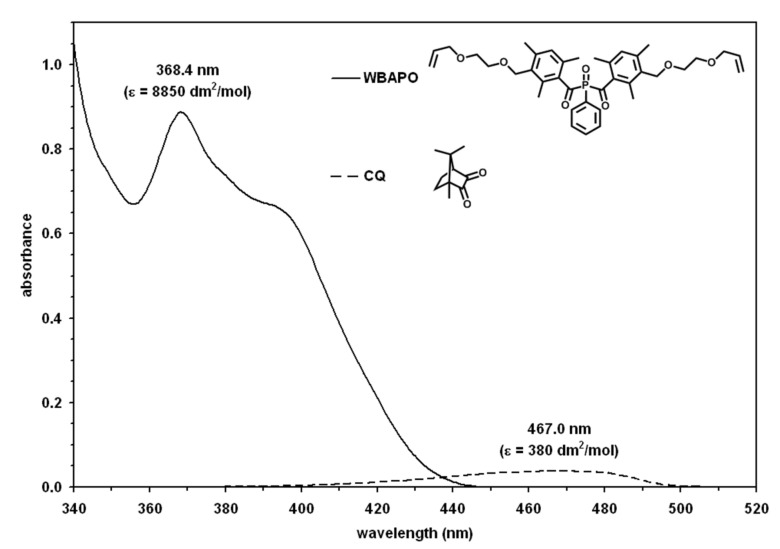
UV–vis absorption spectra of WBAPO and CQ dissolved in acetonitrile (10^−3^ mol/L).

WBAPO is a liquid that exhibits improved solubility in polar solvents compared to the solid BAPO. For example, the solubility of WBAPO in ethanol is about 50% and in acetone >50% compared to the solubility of BAPO of 3% in ethanol or 13% in acetone. As demonstrated in Figure 2, WBAPO dissolved in acetonitrile shows UV–vis absorption, which tails out into the visible range of the spectrum. WBAPO showed almost the same long wavelength absorption maximum (λ_max_) of 368 nm and extinction coefficient (ε) of 8850 dm^2^/mol compared to BAPO (λ_max_ = 369 nm, ε_369_ = 8820 dm^2^/mol). The long wavelength bis(benzoyl)phosphine oxide absorption between ~360 and 400 nm can be generally assigned to symmetry forbidden n-π* transitions, which are responsible for α-cleavage and formation of free radicals [17]. After excitation with light in the near UV–vis, the excited triplet state undergoes cleavage of the carbon-phosphorus bond, thereby producing two highly efficient initiating radicals: a benzoyl and a phosphinoyl radical. CQ absorbs light in the region of 400–500 nm with a low absorption coefficient due to the n-π* transitions of the dicarbonyl group.

**Figure 3:**
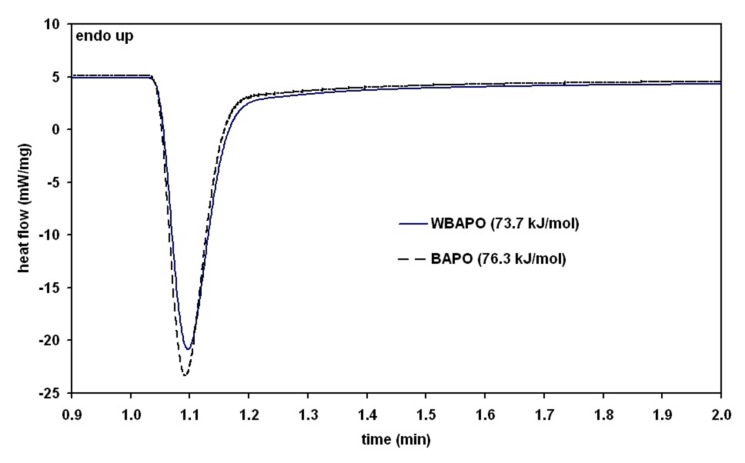
DSC-plot of a mixture of Bis-GMA (42 wt %), UDMA (37 wt %), TEGDMA (21 wt %) and the PI WBAPO or BAPO.

Photo-DSC is a unique method for comparing the performance of different PIs. Therefore, the photopolymerization of a common dental dimethacrylate resin based on mixtures of Bis-GMA (42 wt %), UDMA (37 wt %), TEGDMA (21 wt %) and the PI WBAPO or BAPO (2.38 mmol/100 g resin) was studied by photo-DSC using a blue LED (emission spectrum: 380–515 nm, λ_max_ = 460 nm) as irradiation source. The Photo-DSC plots (Figure 3) confirmed the same photoinitiating activity of the two PIs taking into consideration the experimental accuracy of the DSC method.

Because of the excellent performance of the synthesized WBAPO, it has been used as part of the PI system in our current SEA AdheSE*^®^* One F. This self-etching enamel-dentin adhesive is mainly based on an aqueous mixture of the hydrolytically stable cross-linker DEBAMP and the strongly acidic adhesive monomer BMAMHP. For the investigation of the adhesive properties, the shear bond strength of corresponding compositions containing different PIs was measured as a function of storage time of the adhesive at 42 °C. The results (Table 1) showed that the efficiency of the CQ-A based adhesive decreased very rapidly. In contrast, the bonding properties of the adhesives based on the bisacylphosphine oxide WBAPO or DBDEG were not influenced by the stress test.

**Table 1:** Shear bond strength (SBS, MPa) on dentin of experimental aqueous SEAs^a^ measured after storage of different SEAs at 42 °C.

PI	SBS after 0 d	SBS after 14 d	SBS after 28 d

CQ/EMBO	17.0 ± 4.7	9.7 ± 3.9	n.m.^b^
WBAPO	30.3 ± 4.8	29.4 ± 3.2	30.3 ± 2.7
DBDEG	28.1 ± 3.3	32.2 ± 3.2	29.2 ± 1.6

^a^SEA based on an aqueous mixture of the cross-linker DEBAMP (43 wt %), the strongly acidic monomer BMAMHP (14 wt %) and the different PIs (0.5 wt %); ^b^not measurable.

## Conclusion

WBAPO was synthesized via the dichlorophosphine route in a satisfactory yield. Benzyl and allyl groups of the introduced [2-(allyloxy)ethoxy]methyl substituent probably initiate side reactions during the lithiation step and have a negative effect on the yield of the synthesis. WBAPO, a yellow liquid, showed the expected improved solubility in polar solvents and the same photochemical properties as the commercially available bisacylphosphine oxides. Given its sufficient storage stability, WBAPO can be used as efficient PI in dental acidic aqueous primer formulations.

## Experimental

2,4,6-Trimethylbenzoic acid (Jiangsu Panoxi Chemical Co., Ltd., China) and 2-(allyloxy)ethanol (Kowa Europe GmbH, Germany) were used without further purification. All other substances were purchased from Sigma-Aldrich (Switzerland). 3-(Chloromethyl)-2,4,6-trimethylbenzoic acid was prepared from 2,4,6-trimethylbenzoic acid by chloromethylation according to the literature [15]. DBDEG was synthesized as described previously [18].

DL-camphorquinone (CQ, Rahn, Switzerland), ethyl *p*-dimethylaminobenzoate (EMBO, Fluka Chemie AG, Switzerland) and Irgacure^®^ 819 (BAPO, Ciba Specialty Chemical, Switzerland) were used without purification. Bis-GMA and TEGDMA (Esschem, USA) and 1,6-bis(2-methacryloyloxyethoxycarbonylamino)-2,2,4-trimethylhexane (UDMA, Ivoclar Vivadent AG, Liechtenstein) were purchased from the suppliers noted above.

### Synthesis of **1**

A suspension of 3-(chloromethyl)-2,4,6-trimethylbenzoic acid (84.0 g, 0.395 mol) in 2-(allyloxy)ethanol (200.0 g, 1.96 mol) was added to a solution of potassium hydroxide (88.6 g, 1.58 mol) in 2-(allyloxy)ethanol (606.7 g, 5.94 mol) and heated to 70 °C for 1 h. The mixture was poured in ice-water (600.0 g) and acidified with concentrated hydrochloric acid (pH < 1). After extraction with toluene (2 × 200 mL), the combined organic layers were washed with water (5 × 200 mL), dried with anhydrous sodium sulfate, and the solvent evaporated (50 °C, 80 mbar). Recrystallization of the crude product from cyclohexane yielded **1** as an off-white powder (68.5 g, 62%, mp: 80–81 °C) which was used without further purification.

^1^H NMR (CDCl_3_, 400 MHz): δ (ppm): 2.34, 2.38, and 2.43 (3 s, 3H each, CH_3_), 3.61–3.68 (m, 4H, O(CH_2_)_2_), 4.02 (dt, *J* = 5.3 Hz, 1.4 Hz, 2H, OCH_2_ allyl), 4.60 (s, 2H, OCH_2_ benzyl), 5.15–5.30, and 5.86–5.96 (2 m, 2H, 1H, HC=CH_2_), 6.90 (s, 1H, =CH aromat), 11.0 (br. s, 1H, OH). ^13^C NMR (CDCl_3_, 100 MHz): δ (ppm): 16.7, 19.7 and 19.8 (CH_3_), 67.1, 69.4, 69.7, and 72.2 (OCH_2_), 117.1 (=CH_2_), 129.9 (=CH allyl), 132.0, 132.1, 134.5, 135.0, and 139.6 (=C aromat), 134.6 (=CH aromat), 175.4 (C=O). IR (diamond ATR): ν = 3100 (vbr, OH), 3010 (w, =CH), 2927 and 2878 (m, CH_2_, CH_3_), 1712 (vs, C=O), 1660 (w, C=C allyl), 1604 (w, C=C aromat), 1439 (m, CH_2_, CH_3_), 1352 (m, CH_3_), 1040 (vs, COC), 995 and 923 cm^−1^ (s, =CH allyl). Anal. calcd. for C_16_H_22_O_4_: C, 69.04; H, 7.97. Found: C, 69.17; H, 7.95.

### Synthesis of **2**

**1** (52.3 g, 0.188 mol) was suspended in a mixture of anhydrous toluene (300 mL) and *N*,*N*-dimethylformamide (1.4 mL). Thionyl chloride (33.5 g, 0.282 mol) was added at room temperature. After 2 h, toluene was distilled off and a stream of nitrogen was bubbled through the crude product for 4 h before it was purified by vacuum distillation (130 °C, 0.05 mbar) to give **2** as a colorless liquid (42.6 g, 76%).

^1^H NMR (CDCl_3_, 400 MHz): δ (ppm): 2.33, 2.39, and 2.43 (3 s, 3H each, CH_3_), 3.61–3.68 [m, 4H, O(CH_2_)_2_], 4.02 (d, *J* = 5.3 Hz, 2H, OCH_2,_ allyl), 4.57 (s, 2H, OCH_2_ benzyl), 5.16–5.29, and 5.86–5.96 (2 m, 2H, 1H, HC=CH_2_), 6.91 (s, 1H, =CH aromat). ^13^C NMR (CDCl_3_, 100 MHz): δ (ppm): 16.5, 19.1, and 19.8 (CH_3_), 66.8, 69.67, 69.69, and 72.2 (OCH_2_), 117.1 (=CH_2_), 130.1 (=CH allyl), 132.2, 132.6, 132.7, 138.1 and 140.5 (=C aromat), 134.7 (=CH aromat), 171.0 (C=O). IR (diamond ATR): ν = 3080 (w, =CH), 2980–2861 (m, CH_2_, CH_3_), 1786 (vs, C=O), 1647 (w, C=C allyl), 1599 (w, C=C aromat), 1449 (m, CH_2_, CH_3_), 1348 (m, CH_3_), 1095 (vs, COC), 993, and 924 (=CH allyl), 785 cm^−1^ (vs, CCl). Anal. calcd. for C_16_H_21_ClO_3_: C, 64.75; H, 7.13; Cl, 11.95. Found: C, 64.47; H, 7.15; Cl, 12.42.

### Synthesis of WBAPO

#### 1. Phenylphosphine dilithium

A solution of *P*,*P*-dichlorophenylphosphine (6.59 g, 36.8 mmol) in anhydrous tetrahydrofuran (THF, 10 mL) was added to a stirred mixture of lithium (1.53 g, 221 mmol), naphthalene (0.064 g, 0.50 mmol) and anhydrous THF (40 mL) in a flame dried flask under an argon atmosphere at room temperature. After 22 h, the dark green solution was transferred into another flame dried flask flushed with dry argon via double ended needle. This solution was directly used for the acylation step.

#### 2. Bis(3-{[2-(allyloxy)ethoxy]methyl}-2,4,6-trimethylbenzoyl)(phenyl)phosphine

**2** (21.98 g, 74.1 mmol) was dissolved in anhydrous THF (25 mL). This solution was added dropwise to the above prepared phenylphosphine dilithium solution while the temperature was kept between 30–35 °C in the dark, and subsequently stirred at ambient temperature. After 4 h, THF was removed at 40 °C under reduced pressure and the residue was used in the subsequent oxidation step.

#### 3. Bis(3-{[2-(allyloxy)ethoxy]methyl}-2,4,6-trimethylbenzoyl)(phenyl)phosphine oxide

For this stage all manipulations were carried out in brown glass apparatus or under yellow light. The residue from the latter step was dissolved in toluene (40 mL). Hydrogen peroxide solution (30%, 4.17 g, 36.8 mmol) was added dropwise under vigorous stirring while the temperature was kept at about 70 °C. After the solution was stirred at room temperature for 30 min, it was diluted with ethyl acetate (30 mL). The two layers were separated and the organic layer washed with 0.5 N sodium hydroxide solution (5 × 20 mL) and once with brine (20 mL). After drying with anhydrous sodium sulfate, the solvent was evaporated and the crude product purified by column chromatography (silica gel 60, *n*-heptane:ethyl acetate = 2:1 → 1:2) to give WBAPO as a yellow oil (15.9 g, 67%).

^1^H NMR (400 MHz, CDCl_3_): δ (ppm): 2.10, 2.19, and 2.34 (3 s, 6H each, CH_3_), 3.51–3.56 [m, 8H, O(CH_2_)_2_], 3.98 (dt, *J* = 5.6 Hz, 1.4 Hz, 4H, OCH_2_ allyl), 4.49 (s, 4H, OCH_2_ benzyl), 5.13–5.27, and 5.83–5.93 (2 m, 4H, 2H, HC=CH_2_), 6.84 (s, 2H, =CH aromat), 7.37–7.42, 7.49–7.54 and 7.84–7.88 (3 m, 2H, 1H, 2H, =CH phenyl). ^13^C NMR (100 MHz, CDCl_3_): δ (ppm): 17.1, 19.4 and 19.9 (CH_3_), 66.5, 69.2, 69.6, and 72.2 (OCH_2_), 117.0 (=CH_2_), 125.8 (d, *J*_C-P_ = 74 Hz, =CP), 128.5 (d, ^2^*J*_C-P_ = 11 Hz, =CH), 130.7 (=CH allyl), 132.1 (d, ^3^*J*_C-P_ = 8 Hz, =CH), 132.6, 134.8, 135.5 and 141.2 (=C aromat), 132.9 (d, ^4^*J*_C-P_ = 3 Hz, =CH), 134.7 (=CH aromat), 136.7 (d, ^2^*J*_C-P_ = 41 Hz, =C-C=O), 216.2 (d, *J*_C-P_ = 58 Hz, C=O). ^31^P NMR (162 MHz, CDCl_3_): δ (ppm): 6.58. IR (diamond ATR): ν = 3070 (w, =CH), 2930 and 2860 (m, CH_2_, CH_3_), 1735 (m, C=O), 1679 (m, C=C allyl), 1656 and 1596 (m, C=C aromat), 1437 (m, CH_2_, CH_3_), 1373 (m, CH_3_), 1204 (s, P=O), 1096 (vs, COC), 995 and 925 cm^−1^ (s, =CH allyl). Anal. calcd. for C_38_H_47_O_7_P: C, 70.57; H, 7.32; O, 17.32. Found: C, 70.23; H, 7.28; O, 18.06.

### Synthesis of **3**

Compound **3** was prepared from 3-(chloromethyl)-2,4,6-trimethylbenzoic acid (21.27 g, 0.10 mol) and thionyl chloride in the same way as described for **2**. After vacuum distillation (85–86 °C, 0.1 mbar), **3** was obtained as colorless crystals (14.0 g, 61%, mp: 201–202 °C, decomp.).

^1^H NMR (400 MHz, CDCl_3_): δ (ppm): 2.36, 2.42 and 2.46 (3 s, 3H each, CH_3_), 4.62 (s, 2H, ClCH_2_), 6.95 (s, 1H, =CH). ^13^C NMR (100 MHz, CDCl_3_): δ (ppm): 16.2, 19.2 and 19.4 (CH_3_), 40.1 (ClCH_2_), 130.4 (=CH), 132.1, 132.4, 133.0, 138.2 and 140.0 (=C), 170.3 (C=O). IR (diamond ATR): ν = 3070 (w, =CH), 2980, 2910 and 2870 (w, CH_2_, CH_3_), 1791 (vs, C=O), 1596 and 1566 (m, C=C), 1423 (m, CH_2_, CH_3_), 1381 (s, CH_3_), 772 cm^–1^ (vs, C-Cl). Anal. calcd. for C_11_H_12_Cl_2_O: C, 57.17; H, 5.23; Cl, 30.68. Found: C, 57.56; H, 5.46; Cl, 29.82.

### Synthesis of **4**

To a solution of 2-(allyloxy)ethanol (5.11 g, 50.0 mmol) and triethylamine (5.06 g, 50.0 mmol) in anhydrous methylene chloride (30 mL), **3** (11.56 g, 50.0 mmol) in anhydrous methylene chloride (20 mL) was added at 0 °C. After 2 h the white precipitate was filtered off and the filtrate was washed with water (3 × 50 mL), dried with anhydrous sodium sulfate and the solvent removed under vacuum to give **4** as a colorless liquid (11.3 g, 76%). Compound **4** was also obtained by heating **2** at 180 °C for 4 h and was the main component of its distillation residue.

^1^H NMR (400 MHz, CDCl_3_): δ (ppm): 2.27, 2.36 and 2.38 (3 s, 3H each, CH_3_), 3.72–3.75 (m, 2H, OCH_2_ ether), 4.01–4.03 (m, 2H, OCH_2_ allyl), 4.48–4.50 (m, 2H, OCH_2_ ester), 4.62 (s, 2H, ClCH_2_), 5.16–5.30, and 5.84–5.94 (2 m, 2H, 1H, HC=CH_2_), 6.90 (s, 1H, =CH aromat). ^13^C NMR (100 MHz, CDCl_3_): δ (ppm): 16.2, 19.3 and 19.5 (CH_3_), 40.7 (ClCH_2_), 63.9, 67.8, and 72.02 (OCH_2_), 117.3 (=CH_2_), 130.0 (=CH allyl), 131.9, 133.2, 134.3, 135.2, and 138.7 (=C aromat), 134.4 (=CH aromat), 170.0 (C=O). IR (diamond ATR): ν = 3090 (w, =CH), 2949, 2919 and 2862 (m, CH_2_, CH_3_), 1722 (vs, C=O), 1647 (w, C=C allyl), 1603 (m, C=C aromat), 1447 (s, CH_2_, CH_3_), 1379 (m, CH_3_), 1044 (vs, COC), 992, and 924 cm^−1^ (s, =CH allyl). Anal. calcd. for C_16_H_21_ClO_3_: C, 64.75; H, 7.13; Cl, 11.95. Found: C, 64.83; H, 7.12; Cl, 11.86.

### Measurements

NMR spectroscopic measurements were recorded on a DPX-400 spectrometer (Bruker Biospin, ^1^H: 400 MHz, ^13^C: 100 MHz, ^31^P: 162 MHz) in CDCl_3_ or dimethyl sulfoxide-*d*_6_ as the solvent using tetramethylsilane (TMS) as standard. A FT-IR spectrometer 1600 (Perkin-Elmer) was used to record IR spectra. Melting points were measured with a Melting Point B-540 (Büchi). Elemental analyses were performed with an elemental analyzer CHNS-O Typ EA 1108 (Fisons Instruments). UV–vis spectra were recorded with a UV–vis spectrometer Lambda 2 (Perkin Elmer) in acetonitrile.

The photopolymerization of a dimethacrylate resin (Bis-GMA: 42 wt %, UDMA: 37 wt % and TEGDMA 21 wt %) was studied by photo-DSC (Perkin Elmer DSC 7) using a blue LED Bluephase (1200 mW·cm^−2^, Ivoclar Vivadent AG) and an irradiation time of 180 s at 37 °C.

For the measurement of the shear bond strength of the corresponding dentin adhesives, freshly extracted bovine lower incisors were embedded in unsaturated polyester resin (Castolite, Buehler, USA) in cylindrical molds. Flat dentinal surfaces were ground with water-cooled P240-grit SiC followed by P1000-grit SiC abrasive paper to expose the middle dentin of the embedded teeth. After application of the SEA, solvent evaporation, and light activation, a Teflon mould with a cylindrical hole (3.00 mm in diameter and 4 mm in height, Guillotine method [19]) was placed on the top of bonded surface and filled with two increments of the hybrid composite Tetric Ceram (Ivoclar Vivadent AG). The increments were light cured using a halogen lamp Astralis 10 (emission spectrum: 380–510 nm, 1000 mW·cm^−2^, Ivoclar Vivadent AG) for 40 s each and the test specimens were immersed in water at 37 °C for 24 h prior to testing. Then the shear bond strength was measured using a universal testing machine (Zwick Z010, Germany) at a cross-head speed of 1.0 mm/min.
